# Case Report: Application of the LVIS stent as a bridging device for salvage treatment of malapposed lattice flow diverter in a giant posterior circulation aneurysm: technical note and clinical efficacy

**DOI:** 10.3389/fsurg.2025.1732753

**Published:** 2026-01-20

**Authors:** Musheng Rao, Guan Lin, Shuzhou Cai

**Affiliations:** 1 Department of Neurosurgery, The Central Hospital of Xiaogan, Xiaogan Hospital Affiliate to Wuhan University of Science and Technology, Hubei, China; 2 Department of Neurology, The Central Hospital of Xiaogan, Xiaogan Hospital Affiliate to Wuhan University of Science and Technology, Hubei, China

**Keywords:** bridging technique, flow diverter, incomplete stent expansion, LVIS stent, posterior circulation aneurysm

## Abstract

**Objective:**

To evaluate the feasibility, technical nuances, and clinical outcomes of using the LVIS stent as a bridging device for the salvage treatment of a malapposed Lattice flow diverter (FD) in a giant posterior circulation aneurysm.

**Methods:**

We present a detailed case report of a patient with a giant aneurysm in the V4 segment of the vertebral artery. Following implantation of a Lattice blood flow diverter and coils, immediate post-procedural angiography revealed incomplete opening and malapposition at the proximal segment of the stent, accompanied by delayed distal flow. After unsuccessful attempts to improve wall apposition via microcatheter massage, a salvage strategy was employed by deploying an LVIS stent within the malapposed FD segment. This approach aimed to enhance overall wall apposition and metal coverage to achieve ultimate aneurysm occlusion.

**Results:**

The salvage procedure was performed successfully. The LVIS stent was accurately deployed within the malapposed segment of the FD. Angiographic assessment after the procedure demonstrated complete wall apposition of the composite stent construct and total occlusion of the aneurysm sac. The patient experienced no new neurological deficits during the perioperative period. Short-term follow-up indicated an excellent clinical outcome, with a modified Rankin Scale score of 0.

**Conclusion:**

Utilizing the LVIS stent as a bridging salvage strategy is a safe and effective technical option for managing malapposed FDs in complex giant posterior circulation aneurysms. This technique effectively enhances stent wall apposition and structural integrity, potentially promoting intra-aneurysmal thrombosis and eventual occlusion. It provides a valuable clinical approach for managing this challenging complication.

## 1 Case presentation

**Figure 1:**
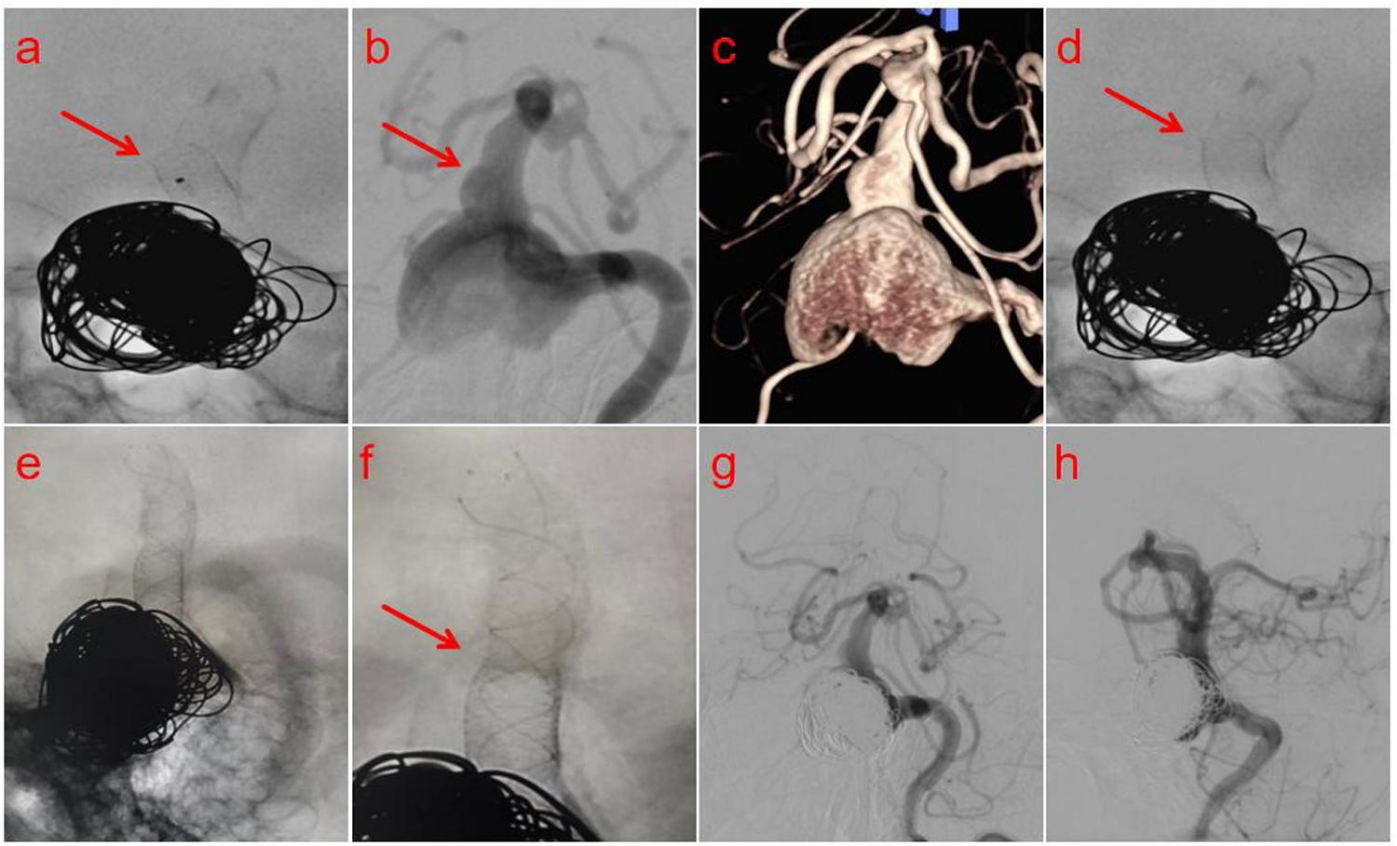
**(a)** Brain MRI revealed a round-like, space-occupying lesion in the left vertebral artery region with significant mass effect upon the adjacent brainstem. **(b,c)** Computed tomography angiography (CTA) of the brain demonstrated an aneurysm in the V4 segment of the left vertebral artery. **(d,e)** Digital subtraction angiography (DSA) confirmed a giant aneurysm of the left vertebral artery. **(f)** 3D vascular reconstruction revealed an aneurysm measuring 26.1 mm × 23.4 mm. **(g)** The first coil was delivered into the aneurysm sac and successfully formed the initial frame. **(h)** Ten coils were deployed into the aneurysm, resulting in complete sealing of the neck.

**Figure 2:**
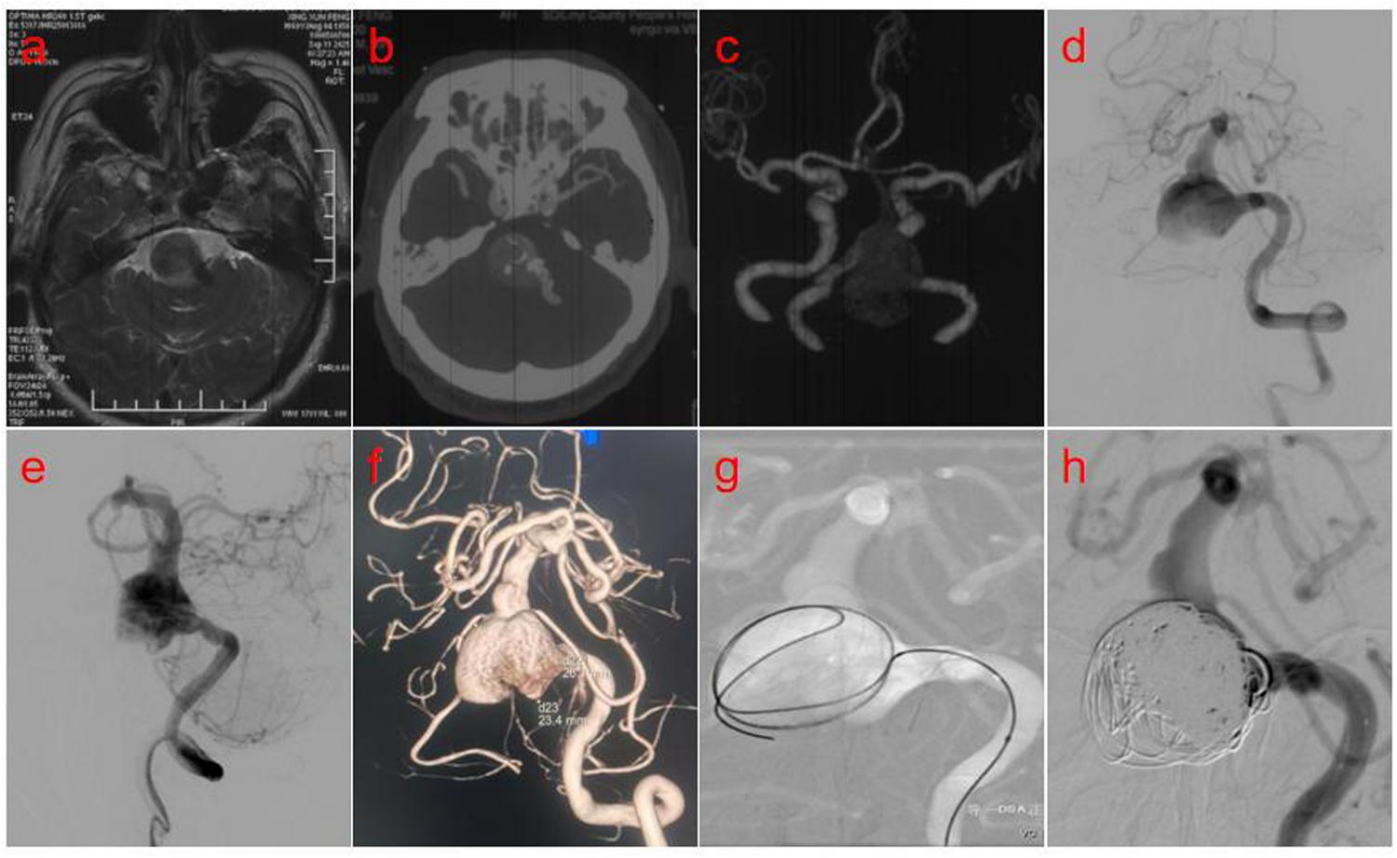
**(a)** Post-deployment angiography identified buckling and wall-ward displacement of the stent's distal end. **(b,c)** Vessel wall ectasia distal to the aneurysm compromised stable anchoring, causing suboptimal stent deployment. **(d)** Mechanical massage with a microguidewire yielded a partial but suboptimal correction of the displaced stent. **(f)** Final angiography demonstrated secure anchoring at the junction of the Lattice and LVIS stents, with both stents in an ideal position, fully expanded, and exhibiting excellent wall apposition. **(g,h)** Standard anteroposterior and lateral projection angiography demonstrated dense embolization of the aneurysm with no evidence of contrast filling, while confirming the patency of the parent artery and the distal posterior circulation flow.

A 69-year-old male patient was admitted to our hospital due to “headache accompanied by weakness in both lower limbs for half a month.” A cranial magnetic resonance imaging (MRI) scan performed at an external hospital revealed a round-like, space-occupying lesion in the region of the left vertebral artery, which exhibited short T1 and T2 signals. Significant compression of the adjacent brainstem and narrowing of the fourth ventricle were noted (Figure 1a). To further clarify the diagnosis, computed tomography angiography (CTA) of the brain was performed, suggesting an aneurysm in the V4 segment of the left vertebral artery (Figures 1b,c). After admission, digital subtraction angiography (DSA) with superselective angiography of the left vertebral artery confirmed the presence of a giant aneurysm of the left vertebral artery (Figures 1d,e). Three-dimensional vascular reconstruction demonstrated the aneurysm size to be approximately 26.1 mm × 23.4 mm (Figure 1f). Following a multidisciplinary team (MDT) discussion, and considering the complex structure and critical location of the aneurysm, the decision was made to perform flow-diverting stent-assisted coil embolization for this posterior circulation aneurysm. The patient received dual antiplatelet therapy (DAPT) (Aspirin 100 mg/day + Clopidogrel 75 mg/day) for 5 days preoperatively. Under general anesthesia, the right femoral artery was successfully punctured using the Seldinger technique, and a 6F arterial sheath was introduced. A 6F guiding catheter was superselected into the V2 segment of the left vertebral artery to establish a stable access pathway. Guided by a Synchro 2 microguidewire, an Echelon microcatheter was navigated with its tip positioned within the aneurysm sac. An Axium QC detachable coil (22 mm × 50 cm, 3D shape) was initially deployed to establish the frame (Figure 1g). After satisfactory framing was achieved, an additional 9 coils of the same model were deployed. Post-coiling angiography demonstrated complete occlusion at the aneurysm neck (Figure 1h). Subsequently, guided by the Synchro 2 microguidewire, a Phenom 27 microcatheter was advanced to the distal basilar artery. A Lattice flow-diverting stent (5.6 mm × 39 mm) was then deployed across the aneurysm neck via this microcatheter. However, post-deployment angiography revealed buckling and displacement of the stent's distal end toward the vessel wall (arrow, Figure 2a), accompanied by delayed opacification of the distal vasculature. Review of the angiographic images indicated that the complication was likely due to the presence of focal vessel wall ectasia distal to the aneurysm (arrows, Figures 2b,c), which compromised stable distal anchoring and optimal stent deployment. This constituted the serious intraoperative complication of incomplete stent opening. Immediate attempts were made to reposition the displaced stent segment using microcatheter massage. The stent position was partially improved (arrow, Figure 2d), but persistent incomplete apposition of the distal end remained despite repeated maneuvers. Consequently, a bridging technique with a braided stent was employed: an LVIS stent (5.5 mm × 30 mm) was deployed, partially overlapping with the flow-diverting stent and bridging the segment distal to the aneurysm (Figure 2e). Final control angiography confirmed secure anchoring at the junction between the Lattice and LVIS stents (arrow, Figure 2f), with both stents in an ideal position, fully expanded and exhibiting good wall apposition. Standard anteroposterior and lateral projections demonstrated dense aneurysm embolization with no contrast filling, and patency of the parent artery and distal posterior circulation blood flow (arrows, Figures 2g,h). Postoperatively, the patient received an intravenous infusion of Tirofiban for 24 h, which was subsequently transitioned to dual antiplatelet therapy with Aspirin (100 mg once daily) and Ticagrelor (90 mg every 12 h). At the 1-month follow-up, the patient was recovering well with no new neurological deficits.

## 2 Discussion

The management of intracranial aneurysms remains a significant challenge in the field of neurosurgery, with giant posterior circulation aneurysms posing particular difficulty. Located in critical intracranial anatomical regions and adjacent to complex structures, these aneurysms are not only technically demanding to address via traditional craniotomy but also often preclude ideal neck clipping. Regarding endovascular treatment, while simple coil embolization is widely used, it frequently fails to completely eliminate the inflow jet into the aneurysm sac, thereby inadequately promoting endothelialization and healing at the neck. Furthermore, the large size and irregular morphology of giant posterior circulation aneurysms often complicate stable coil packing, predisposing to coil migration, compaction, or even protrusion, which leads to incomplete occlusion and higher long-term recurrence rates. Even with stent-assisted coil embolization techniques, the wall tension of the aneurysm is not sufficiently alleviated, failing to fundamentally eliminate the risk of re-rupture. In recent years, advancements in endovascular interventional techniques have introduced the flow diverter (FD) as a novel approach for managing these challenging aneurysms (1). The FD, a high-density mesh stent deployed across the aneurysm neck, significantly alters local hemodynamics by reducing inflow into the sac while maintaining patency of the parent artery. This device not only mitigates the risk of rupture but also promotes the migration and growth of endothelial cells along its scaffold, facilitating reconstruction and repair of the vessel wall at the neck. Over time, intra-aneurysmal blood flow stagnates, leading to progressive thrombosis and eventual anatomical cure of the aneurysm (2).

While flow diverters (FDs) demonstrate significant advantages in the treatment of intracranial aneurysms, their clinical application is accompanied by a spectrum of complication risks. These primarily include incomplete device expansion, in-stent thrombosis, and vascular perforation or rupture. In-stent thrombosis represents a serious complication (3), often associated with inadequate antiplatelet therapy or a patient's inherent hypercoagulable state. Thrombus formation can lead to acute occlusion of the parent artery or its distal branches, precipitating cerebral ischemic symptoms in the corresponding territory, which may severely progress to cerebral infarction. Vascular perforation or rupture is a more critical emergency. It typically occurs due to improper manipulation of the guidewire, microcatheter, or the stent delivery system during deployment, or in cases where the patient's vessel wall is inherently fragile or diseased. This can result in intraprocedural acute subarachnoid hemorrhage, threatening the patient's life (4). Furthermore, incomplete FD expansion is frequently encountered in clinical practice (5). If the stent fails to fully expand, it leads to poor wall apposition. This not only compromises the device's intended flow-diverting effect at the aneurysm neck but may also increase the risk of in-stent thrombosis and distal embolism due to altered intra-stent flow dynamics. Given the potential severe impact of these complications on patient outcomes, it is imperative to strictly adhere to indications for FD placement, standardize procedural techniques, and implement rigorous post-operative patient monitoring for early identification and management of related adverse events (6). Among these challenges, the effective prevention and management of incomplete FD expansion has emerged as a critical technical problem requiring urgent optimization and resolution in the field of endovascular intervention.

Incomplete flow diverter (FD) expansion can be attributable to a variety of factors.

### 2.1 Patient-specific vascular anatomical factors

Patient-specific variations in vascular anatomy represent a critical determinant influencing successful stent deployment. Anatomical variations, such as severe tortuosity of the access vasculature, anomalous branching angles, or a target vessel diameter that is excessively small or large, can all increase the difficulty of device navigation and deployment, leading to incomplete expansion or poor wall apposition (7). Consequently, a comprehensive and precise pre-procedural assessment of the cerebrovascular anatomy is mandatory to inform the development of an individualized treatment strategy. Should incomplete expansion occur intraoperatively due to suboptimal vascular conditions, balloon angioplasty may be considered to pre-dilate stenotic or tortuous segments. This intervention can improve access conditions and create a more favorable luminal environment to facilitate complete device expansion.

### 2.2 Intraoperative technical factors

The operator's technical skill and experience level directly influence the deployment outcome of the stent. Inappropriate maneuvers during advancement—such as uneven force application, misalignment of the guidewire direction, or improper control of the deployment rhythm—may result in incomplete expansion of the stent at the target site (8). Therefore, the operator must possess proficient interventional skills and substantial surgical experience, maintaining precision and stability throughout the procedure. Should suboptimal deployment occur, the procedure should be paused immediately to reassess the positions of the guidewire and microcatheter, as well as the status of the stent system. Gentle intra-luminal massage via the microcatheter may be attempted to facilitate wall apposition and expansion of the stent. If these measures prove insufficient, the feasibility of deploying an overlapping additional stent may be evaluated based on the aneurysm morphology and parent artery condition. As a last resort, conversion to surgical intervention should be considered. Any remedial strategy must be cautiously decided by an experienced medical team after comprehensively weighing the potential benefits and risks for the patient.

### 2.3 Device-related performance factors

While the design and manufacturing quality of most contemporary stents are generally reliable, they remain a non-negligible factor influencing deployment. For instance, inherent device limitations such as material defects introduced during manufacturing, structural heterogeneity, or suboptimal delivery system performance can all impede proper *in vivo* expansion (9). Therefore, stringent quality assessment during device selection is imperative, favoring products from brands and models that have undergone rigorous clinical validation and demonstrated stable performance. Should deployment failure occur intraoperatively with a high suspicion of device-related issues, the stent should be promptly replaced to complete the procedure. From a long-term development perspective, medical device companies are encouraged to continually refine the design concepts and manufacturing processes of flow diverters. This includes developing next-generation intelligent stents with enhanced flexibility, improved radiopacity, or adaptive morphology to better conform to vascular anatomy. Such advancements are fundamental to improving device deliverability and deployment success rates, ultimately providing safer and more effective treatment options for complex cases.

The LVIS stent, as a braided closed-cell device, typically provides superior radial force compared to laser-cut stents (10), enabling reliable support to the vessel wall and thereby effectively mitigating issues of incomplete stent expansion and poor wall apposition. Concurrently, its delicate mesh design aids in preserving the patency of branching vessels. In contrast, flow diverters (FDs) primarily function by reconstructing the hemodynamics of the parent artery, significantly reducing inflow into the aneurysm sac, promoting intra-aneurysmal thrombosis and neointimal hyperplasia, ultimately achieving complete aneurysm occlusion (11). The “stent bridging” technique, which involves the combined deployment of an LVIS stent with an FD, integrates the therapeutic advantages of both devices. On one hand, the LVIS stent serves as an internal scaffolding structure, providing mechanical assistance to a malapposed FD, thereby restoring and maintaining the luminal geometry and patency of the parent artery. On the other hand, the FD can further augment the flow-diverting effect on the target aneurysm. This dual mechanism of action is anticipated to significantly improve rates of complete aneurysm occlusion, offering a more reliable treatment strategy for managing complex aneurysms, particularly those involving tortuous anatomy or suboptimal FD deployment.

When employing the LVIS stent bridging technique with a flow diverter (FD), several critical considerations must be addressed. First, a detailed pre-procedural assessment of the patient's vascular anatomy and aneurysm characteristics is essential to select an appropriately sized stent, ensuring a optimal match between the stent and the vessel diameter. Second, intraoperative manipulation must be meticulous, involving precise stent positioning and controlled deployment force to avoid injury to the vessel wall or branch vessels. Third, standardized dual antiplatelet therapy is mandatory postoperatively to prevent in-stent thrombosis, accompanied by close monitoring of the patient's clinical status for timely management of any potential complications. In the present case, immediate post-procedural angiography following the initial deployment of a Lattice flow diverter revealed incomplete expansion at the distal end of the stent, resulting in poor wall apposition and associated delayed opacification of the distal vasculature. To address this technical complication, a remedial strategy was employed: an LVIS stent was deployed as a bridging stent within the lumen of the original FD. Owing to its high metal coverage rate and favorable radial force, the LVIS stent effectively enhanced the overall wall apposition of the composite construct, thereby resolving the initial incomplete expansion of the FD (12, 13). Control angiography confirmed that following the bridge stent placement, stent wall apposition was significantly improved, and the parent artery lumen was restored to patency. Concurrently, the flow-diverting effect was enhanced, promoting intra-aneurysmal thrombosis. Subsequent angiographic evaluation demonstrated radiographic cure of the aneurysm, confirming that this salvage approach achieved a satisfactory clinical outcome. This study was limited to a 1-month follow-up period. Although favorable perioperative outcomes were observed, the long-term efficacy of the aneurysm treatment remains to be validated, which requires confirmation through extended follow-up in future studies.

In conclusion, the utilization of an LVIS stent as a bridging salvage technique represents a safe and effective strategic option for managing cases of incomplete flow diverter (FD) expansion following treatment of giant posterior circulation aneurysms. This approach not only effectively enhances the overall wall apposition of the stent construct and restores hemodynamic stability but also contributes to achieving higher rates of final aneurysm occlusion. The successful outcome in this specific case confirms the clinical feasibility of this strategy, providing a valuable reference for managing similar complex scenarios. Future efforts should focus on accumulating larger case series and obtaining long-term follow-up data to further validate the technique's long-term efficacy and delineate its applicable scope, thereby continuously refining the therapeutic workflow and advancing the standards of endovascular intervention for such complex cerebrovascular pathologies.

## Data Availability

The original contributions presented in the study are included in the article/supplementary material, further inquiries can be directed to the corresponding author/s.
